# Prion pathogenesis is unaltered in a mouse strain with a permeable blood-brain barrier

**DOI:** 10.1371/journal.ppat.1007424

**Published:** 2018-11-29

**Authors:** Annika Keller, Mario Nuvolone, Irina Abakumova, Andra Chincisan, Regina Reimann, Merve Avar, Daniel Heinzer, Simone Hornemann, Josephin Wagner, Daniel Kirschenbaum, Fabian F. Voigt, Caihong Zhu, Luca Regli, Fritjof Helmchen, Adriano Aguzzi

**Affiliations:** 1 Department of Neurosurgery, Clinical Neuroscience Centre, University Hospital Zürich, Zürich University, Zürich, Switzerland; 2 Institute of Neuropathology, University Hospital Zürich, Zürich University, Zürich, Switzerland; 3 Amyloidosis Research and Treatment Center, Foundation IRCCS Policlinico San Matteo, Department of Molecular Medicine, University of Pavia, Pavia, Italy; 4 Brain Research Institute, Zürich University, Zürich, Switzerland; 5 Neuroscience Center Zürich (ZNZ), University of Zürich and ETH Zürich, Zürich, Switzerland; University of Edinburgh, UNITED KINGDOM

## Abstract

Transmissible spongiform encephalopathies (TSEs) are caused by the prion, which consists essentially of PrP^Sc^, an aggregated, conformationally modified form of the cellular prion protein (PrP^C^). Although TSEs can be experimentally transmitted by intracerebral inoculation, most instances of infection in the field occur through extracerebral routes. The epidemics of kuru and variant Creutzfeldt-Jakob disease were caused by dietary exposure to prions, and parenteral administration of prion-contaminated hormones has caused hundreds of iatrogenic TSEs. In all these instances, the development of postexposure prophylaxis relies on understanding of how prions propagate from the site of entry to the brain. While much evidence points to lymphoreticular invasion followed by retrograde transfer through peripheral nerves, prions are present in the blood and may conceivably cross the blood-brain barrier directly. Here we have addressed the role of the blood-brain barrier (BBB) in prion disease propagation using *Pdgfb*^*ret/ret*^ mice which possess a highly permeable BBB. We found that *Pdgfb*^*ret/ret*^ mice have a similar prion disease incubation time as their littermate controls regardless of the route of prion transmission. These surprising results indicate that BBB permeability is irrelevant to the initiation of prion disease, even when prions are administered parenterally.

## Author summary

Prion diseases or transmissible spongiform encephalopathies (TSEs) are incurable brain diseases caused by conformational changes in the endogenous prion protein. Prions can be transmitted through contaminated food, surgical instruments and blood. Transmission of prions has caused the kuru epidemic in humans and bovine spongiform encephalopathy in cattle, which in turn caused variant Creutzfeldt-Jakob disease (CJD) in humans. Furthermore, injection of prion-contaminated hormones has caused hundreds of TSE cases. In order to develop drugs to prevent the spread of prions into the brain after exposure via food or medical procedures, it is necessary to gain an understanding of how prions propagate from the site of entry to the brain. Prions were shown to reach the spinal cord by traveling along peripheral nerves. However, prions are also found in blood. Although normal brain vessels act as a barrier between the blood and brain, some studies suggested that prions in blood may enter the brain via blood vessels. Here we have tested the latter hypothesis using mice with increased brain blood vessel permeability. We found that these mice are similar to wild-type mice in their susceptibility to prion disease and incubation times after peripheral inoculation. These results suggest that passage of prions through the blood-brain barrier may not be relevant to the development of disease, and imply that any effective post-exposure treatment should rather aim at other rate-limiting steps of prion propagation.

## Introduction

Transmissible spongiform encephalopathies (TSEs) are progressive, invariably lethal neurodegenerative diseases which include Creutzfeldt–Jakob disease, kuru, fatal familial insomnia and Gerstmann–Sträussler–Scheinker syndrome in humans, scrapie in sheep, and bovine spongiform encephalopathy (BSE) in cattle [1]. The infectious agent, termed prion, consists primarily of PrP^Sc^, a conformationally modified form of PrP^C^, a protein encoded by the gene *PRNP* [2]. Conversion of PrP^C^ into PrP^Sc^ leads to accumulation of insoluble, partially protease-resistant prion protein deposits in the brain parenchyma around neurons and neuronal loss which is accompanied by gliosis and spongiform changes. Deletion of PrP^C^ renders mice resistant to prion infections, indicating that its conversion into PrP^C^ is necessary for the development of disease [1].

Although there have been instances of patients intracerebrally infected by prion-contaminated medical equipment or by dura mater grafts of cadaveric origin [3], transmission of prion infections occurs more frequently through peripheral routes. The oral route of transmission has caused epidemics of kuru and variant CJD in humans, as well as BSE in cows [3]. Likewise, the parenteral route of prion transmission is highly effective in laboratory mice and hamsters.

But how do prions reach the central nervous system (CNS) upon entering the body from peripheral sites? After extraneural inoculation, prions accumulate and replicate in lymphoid tissues [4]. Follicular dendritic cells (FDC) and their precursors may constitute the first site of prion amplification [1]. Several studies indicate that prions travel to the CNS along peripheral sympathetic nerves, and the distance between FDC and sympathetic nerve endings specifies the speed of neuroinvasion [5, 6].

However, none of these findings exclude the possibility that prions, in addition to following the lymph invasive route, may directly colonize the CNS through hematogenic spread followed by direct crossing of the brain vasculature. Prions are present in the blood of hamsters, mice and humans, and it was recently shown that both, PrP^c^ and PrP^Sc^, can cross the blood-brain barrier (BBB) [7–9]. Whether this contributes to the initial spread of the disease into the CNS is largely unknown.

Here we have addressed the role of the BBB in prion pathogenesis using a genetically modified mouse strain (*Pdgfb*^*ret/ret*^) which possesses a highly-permeable BBB as the result of the expression of a platelet-derived growth factor B (PDGF-B) lacking the PDGF-B retention motif [10, 11]. We show that *Pdgfb*^*ret/ret*^ mice succumb to prion disease similarly to their littermate controls regardless of the route of prion transmission. In addition, there are no differences in histopathological characteristics of the disease nor in the resistance to the protease K of PrP^Sc^ in the brains of terminally sick *Pdgfb*^*ret/ret*^ mice compared to the controls. Our study indicates that although PrP^Sc^ can cross the BBB [7, 8], this route of entry into the CNS is negligible as regards the initiation of the disease when prions are administered intravenously, and highlights the importance of peripheral replication in prion disease pathogenesis in the case of blood-borne transmission.

## Material and methods

### Animals

*B6*.*129-Pdgfb<tm3Cbet>* [12] heterozygous mice (*Pdgfb*^*wt/ret*^) in the C57BL/6J genetic background were crossed to obtain *Pdgfb*^*wt/wt*^, *Pdgfb*^*wt/ret*^ and *Pdgfb*^*ret/ret*^ littermates that were used for prion infection studies. *Pdgfb*^*ret/ret*^ animals possess an open BBB [10]. The BBB defect occurs at the level of endothelial transcytosis and tracers with a wide range in the molecular weight (1 kDa– 200 kDa) or different chemical composition enter the brain parenchyma in *Pdgfb*^*ret/ret*^ animals.

### Ethics statement

Animal care and experimental protocols were in accordance with the “Swiss Ethical Principles and Guidelines for Experiments on Animals”, and approved by the Veterinary office of the Canton of Zurich (permits ZH130/2008, ZH14/2012, ZH90/2013 and ZH196/2014).

### Blood brain barrier analysis

*Pdgfb*^*wt/ret*^ and *Pdgfb*^*ret/ret*^ mice received 2.5mg/20g 70 kDa-dextran conjugated to Texas Red (Invitrogen, Cat # D1864) via the tail vein. The tracer was allowed to circulate for 5 hours. Mouse brain tissue was prepared for whole-brain clearing according to published protocols [13, 14]. Mice were deeply anaesthetized and transcardially perfused with ice cold PBS followed by a fixative mixture of 4% acrylamide, 1% paraformaldehyde, 0.05% Bis, 0.25% VA-044 in PBS. Mouse brains were removed and post-fixed in the same fixative for 24 hours at 4 °C. The brains were de-gassed, exposed to gaseous nitrogen, and polymerized for 2.5 hours at 37 °C. Brains were extracted from the hydrogel and placed in 8% sodium dodecyl sulfate (SDS), 200 mM boric acid, pH 8.5 (clearing solution). Brains underwent clearing by electrophoresis (4–8 hours). Cleared brains were then washed in PBS. The refractive index was equilibrated with refractive-index matching solution prepared according to published protocols [14]. Brains were imaged using a custom mesoscale selective plane illumination microscope (mesospim.org) that will be described in detail elsewhere. Images were processed using Image J and Imaris (Bitplane) software.

### Prion inoculation

Mice were infected with the Rocky Mountain Laboratory (RML) scrapie strain (passage 6, RML6). Three different inoculation routes were used: intracerebral, intravenous and intraperitoneal. For inoculations, we used 30 μl of RML6 brain homogenate prepared in a solution of 0.32 M sucrose containing 5% BSA. Control groups of mice received intracerebrally 30 μl of non-infectious brain homogenate (NBH, 10% w/v) prepared from healthy CD-1 mice. Clinical assessment and scoring of mice based on the presence of neurological signs (including ataxia, kyphosis, priapism, leg paresis, lack of grooming) was performed as previously described [15]. Mice were euthanized on the day of onset of clinical signs of scrapie according to the approved protocols.

One group of mice was inoculated intracerebrally with 30 μl of RML6 brain homogenate containing 1.5 log LD_50_ of infectious agent. Two groups of mice received RML6 intravenously 100 μl of RML6 brain homogenate containing 6 log LD_50_ and 100 μl of RML6 brain homogenate containing 3 log LD_50_ of infectious agent. One group of mice received 30 μl of RML6 brain homogenate containing 4.5 log LD_50_ intraperitoneally. Prism software (www.graphpad.com) was used to perform statistical analysis. The log-rank test was used to compare the survival curves between *Pdgfb*^*wt/wt*^, *Pdgfb*^*ret/wt*^ and *Pdgfb*^*ret/ret*^ littermates.

### Western blot

Brains were homogenized in 0.32 M sucrose in PBS. Total protein concentration was determined using the bicinchoninic acid assay (Pierce) according to manufacturer’s instructions. Samples were adjusted to 1 μg/μl and digested with proteinase K (PK) (20 μg/μl) in PBS, 0.5% SDS and 0.5% NP-40 for 30 minutes at 37 °C. Proteinase K reaction was stopped by adding loading buffer (Invitrogen) followed by boiling samples for 5 minutes at 95 °C. PK-treated and untreated samples were separated on a 12% Bis-Tris polyacrylamide gel (NuPAGE, Invitrogen) and blotted onto a nitrocellulose membrane. Anti-PrP antibody (POM1, 200 ng ml^−1^) [16] was used as a primary antibody which was detected using rabbit anti-mouse IgG_1_ conjugated to horseradish peroxidase (HRP). Western blots were developed using Luminata Crescendo Western HRP substrate (Millipore) and visualized using the FUJI-FILM LAS-3000 system. The glycoform profiles of PrP^Sc^ after PK treatment were quantified using the Quantify One software (BioRad). The relative intensity of each PrP^Sc^ glycoform (i.e. di-, mono-, ungylcosylated) was measured which was expressed then as a percentage of the total signal. Statistical analysis (two-way ANOVA) was performed using the Prism software (www.Graphpad.com).

### Histochemistry and immunohistochemistry

Formalin-fixed tissues were treated with concentrated formic acid for 60 minutes at room temperature to inactivate prion infectivity. Tissue was embedded in paraffin and cut into 2 μm sections. After deparaffinization through graded alcohols sections were stained with hematoxylin/eosin. Antibody SAF-84 (A03208, 1:200, SPI-Bio, Waterloo, Australia) was used to detect partially protease-resistant prion protein deposition on a NEXES immunohistochemistry robot (Ventana Instruments, Basel Switzerland) using an IVIEW DAB Detection Kit (Ventana), after incubation with protease 1 (Ventana). Microglia was detected using anti-Iba 1 antibody (WAKO). Sections were deparaffinized through graded alcohols and heat-induced antigen retrieval was performed in citrate buffer (0.01 M; pH 6). Sections were incubated with anti-Iba1 Ab (1∶2500). Stainings were visualized using DAB (Sigma-Aldrich) and H_2_O_2_ (Sigma-Aldrich), after incubation with a biotinylated secondary antibody (Vector Laboratories) followed by the ABC complex solution (Vector laboratories). Sections were counterstained with Hematoxylin. Images of HE and DAB stained sections were acquired using a NanoZoomer scanner (Hamamatsu Photonics) and NDPview digital pathology software (Hamamatsu Photonics).

### Quantification of microglia and vacuoles

The quantification of Iba1 positive cells was performed after DAB immunohistochemistry (n = 4 fields/mouse, n = 3–6 mice/group) using Qupath software (manual quantification function) [17]. Data were presented as number of Iba1 positive cells/mm^2^. Quantification of vacuoles was performed on HE images ((n = 8 fields/mouse, n = 3–5 mice/group). The algorithm to count vacuoles was developed in MATLAB R2016B using the Image Processing toolbox. Image segmentation was performed using Otsu’s thresholding method (T = 0.7). Only round vacuoles with an area in the range of [200 pixels, 2000 pixels] and with a shape values > 0.9 (where shape was calculated as shape = (4*π*Area)/(Perimeter^2)) were quantified. The code is available at: https://github.com/AndraCh/Vacuoles_segmentation. Statistical analysis (one-way ANOVA) was performed using the Prism software (www.Graphpad.com).

### Determination of PK-resistant PrP^Sc^ levels by fluorescence-resonance energy transfer (FRET)

PK-resistant PrP^Sc^ was quantified in brain tissue homogenate by FRET using monoclonal antibodies POM1 and POM19 [16]. Protein concentration of samples were determined with a bicinchoninic acid assay performed according to the manufacturer’s instructions (Thermo Fisher Scientific). A total amount of 10 μg protein per well was diluted to the correct volume in PBS. To determine PK-resistant PrP^Sc^ levels, samples were PK digested using 50 μg/ml PK (Roche) at 37°C for 30 min under constant agitation. Digestion was stopped by adding PMSF to a final concentration of 2.24 mM and samples were incubated for 10 min at room temperature (RT). Denaturation of remaining PrP^Sc^ in samples was achieved by the addition of NaOH to a final concentration of 56 mM and samples were incubated for 10 min at RT under constant agitation. To neutralize the samples, NaH_2_PO_4_ was added to a final concentration of 66 mM and incubated for 10 min at RT. Samples were pipetted in triplicates to a 384-well OptiPlate (Perkin Elmer). For FRET assay we used two in-house produced monoclonal antibodies recognizing different epitopes of PrP (POM19 and POM1) [16]. POM19 was coupled to Europium (EU, FRET donor) and POM1 was coupled to allophycocyanin (APC, FRET acceptor). The antibody pair was diluted in 1X Lance buffer (Perkin Elmer) to a final concentration of EU-POM19 of 2.5 nM and APC-POM1 of 5 nM. After adding the antibody pair to samples, the plate was centrifuged at 2000 g for 1 minute and incubated overnight at 4°C. The following day, FRET was measured using a multilabel plate reader (EnVision, Perkin Elmer). The excitation wavelength was 337 nm and emission wavelength for EU was 615 nm and for APC 665 nm. Following measurement, the net-FRET signal was used in accordance with formula published in [18]. For subsequent analysis, the triplicates were averaged and the signal for non-infectious brain homogenate was subtracted to remove the background. PrP^Sc^ levels are presented as a ratio of PrP^Sc^ in each individual animal to the average of wild-type littermates. Statistical analysis (one-way ANOVA) was performed using the Prism software (www.Graphpad.com).

## Results and discussion

In order to detect any possible role of the BBB in prion pathogenesis we used *Pdgfb*^*ret/ret*^ mice which express a hypomorphic variant of PDGFB [10]. The cerebrovascular tree in these animals is highly permeable to blood-borne macromolecules, as has been demonstrated in several studies using many orthogonal experimental approaches including immunohistochemistry, spectrophotometry, MRI [10, 11, 19]. Surprisingly, these mice are viable and enjoy an almost-normal life span despite their deeply dysfunctional BBB. These findings made it possible to perform prion infection experiments and study the development of disease over many months.

Certain areas of the brain are differentially susceptible to neurodegeneration induced by different prion strains [20]. It is conceivable that the BBB defect of *Pdgfb*^*ret/ret*^ mice may not coincide with the areas of selective vulnerability to prions. Therefore, we investigated the regional characteristics of BBB permeability in *Pdgfb*^*ret/ret*^ mice using electrophoretic clearing based on the CLARITY method [13] in custom designed clearing chambers. After receiving an intravenous injection of 70 kDa dextran conjugated to TexasRed, mice were perfused, brains were cleared and then imaged using a mesoscale selective-plane illumination microscope. The entire brain of *Pdgfb*^*ret/ret*^ animals showed passage of 70 kDa-dextran Texas Red into the brain parenchyma (S1B Fig and S1 Movie). The strongest leakage of dextran was detected in the cortex compared to other brain regions, which is in agreement with previously published data [19]. In control animals (*Pdgfb*^*wt/ret*^), no fluorescent signal can be detected in the brain parenchyma (S1A Fig). Thus, these data further demonstrate an enhanced permeability of the BBB in the entire brain of *Pdgfb*^*ret/ret*^ mice.

**Fig 1:**
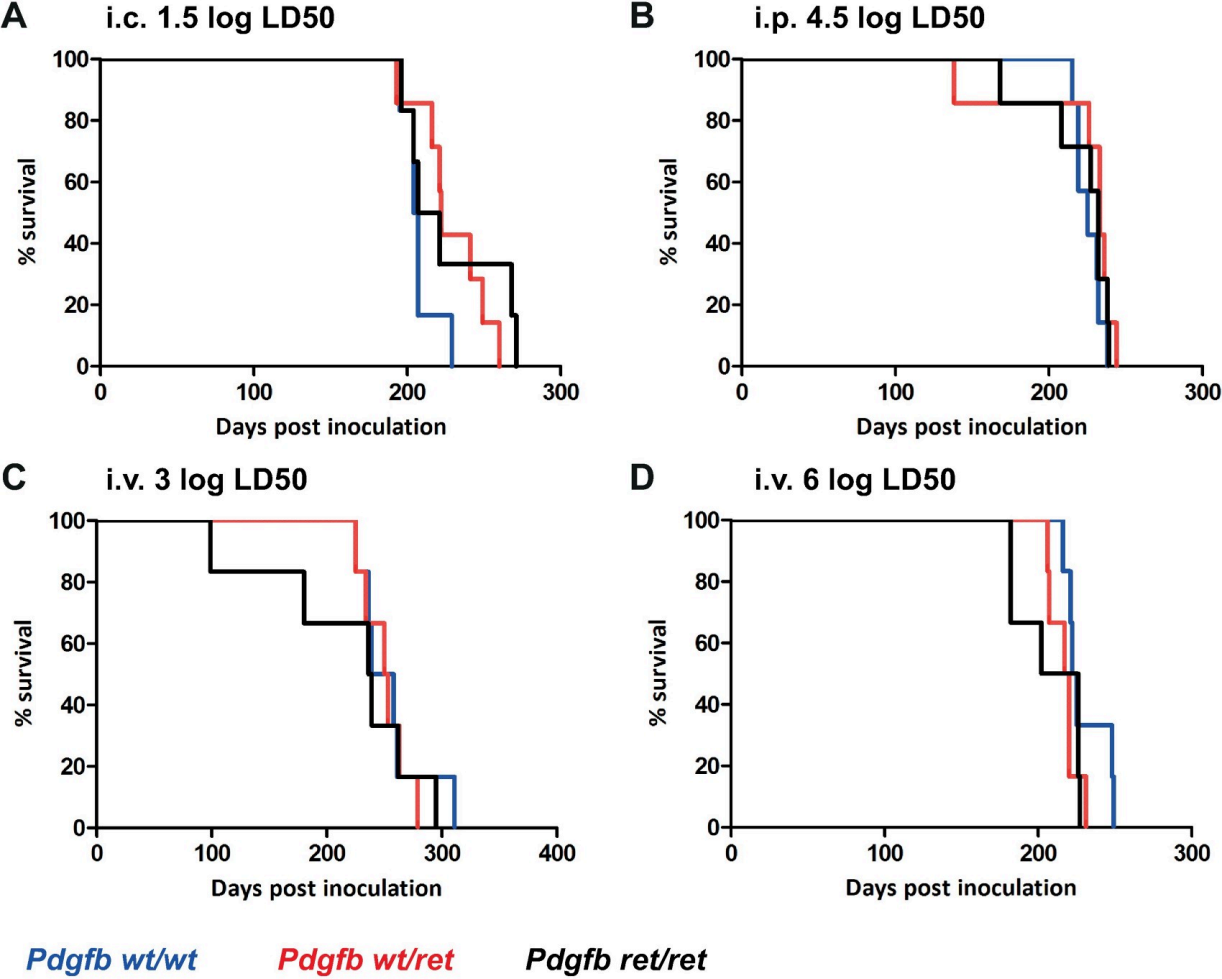
Increased BBB permeability does not alter prion disease incubation. (A-D) Kaplan-Meier survival curves of *Pdgfb*^*wt/wt*^, *Pdgfb*^*wt/ret*^, *Pdgfb*^*ret/ret*^ animals inoculated with RML6 intracerebrally (A) (1.5 log LD_50_ *Pdgfb*^*wt/wt*^ n = 6, 205.5 dpi; *Pdgfb*^*wt/ret*^ n = 7, 222 dpi; *Pdgfb*^*ret/ret*^ n = 6, 214 dpi, P = 0.22), intraperitoneally (B) (4.5 log LD_50_ *Pdgfb*^*wt/wt*^ n = 7, 225 dpi; *Pdgfb*^*wt/ret*^ n = 7, 233 dpi; *Pdgfb*^*ret/ret*^ n = 7, 232 dpi, P = 0.41) or intravenously (C, D) (3 log LD_50_ (C) *Pdgfb*^*wt/wt*^ n = 6, 248.5 dpi; *Pdgfb*^*wt/ret*^ n = 6, 237.5 dpi; *Pdgfb*^*ret/ret*^ n = 6, 237.5 dpi, P = 0.86 and 6 log LD_50_ (D) *Pdgfb*^*wt/wt*^ n = 6, 223.5 dpi; *Pdgfb*^*wt/ret*^ n = 6, 218.5 dpi; *Pdgfb*^*ret/ret*^ n = 6, 214 dpi, P = 0.37).

To test whether *Pdgfb*^*ret/ret*^ and control mice (*Pdgfb*^*wt/wt*^, *Pdgfb*^*wt/ret*^) are competent for prion replication and succumb into prion disease similarly, mice were inoculated intracerebrally with RML6. There was no difference in disease incubation between *Pdgfb*^*ret/ret*^ and control mice (*Pdgfb*^*wt/wt*^, *Pdgfb*^*wt/ret*^) when infected intracerebrally (Fig 1A).

**Fig 2:**
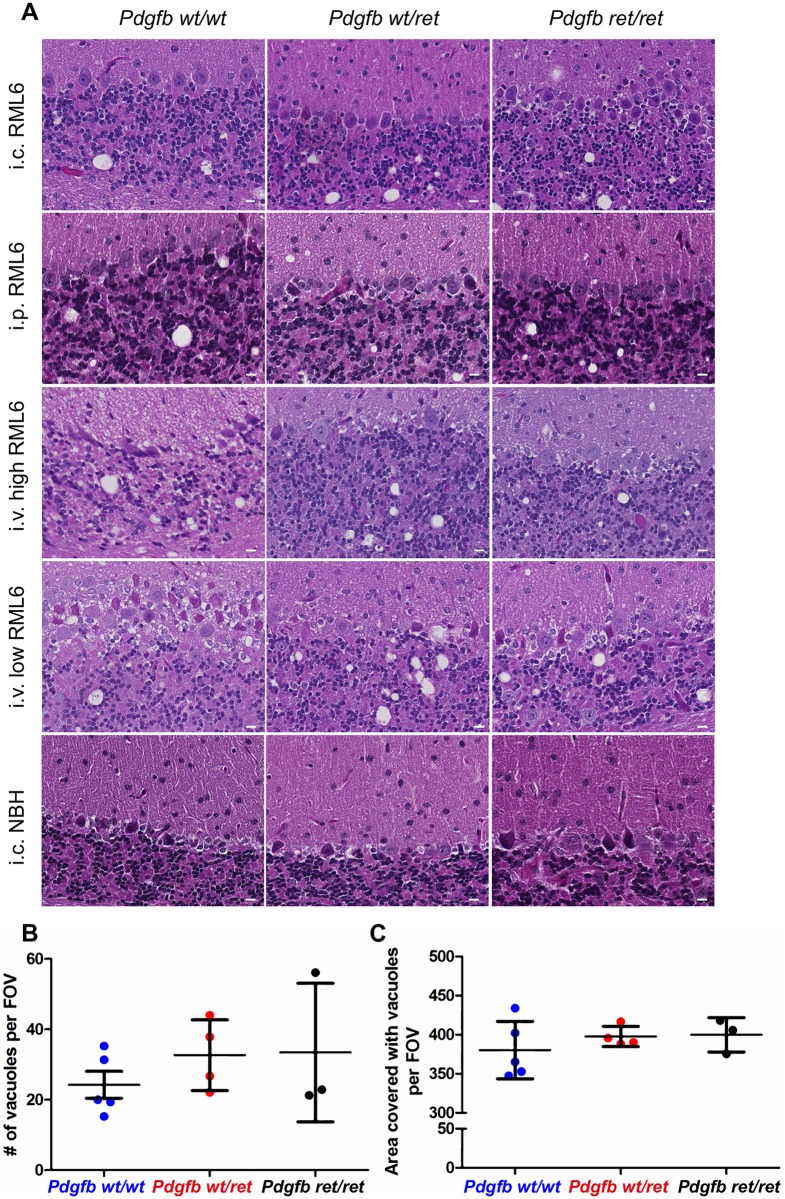
Prion histopathology and vacuolation after RML6 inoculation in control and BBB-compromised mice. (A) Hematoxylin/eosin stained sections from the cerebella of prion-inoculated (RML6) terminally sick (or control, normal brain homogenate (NBH) inoculated mice) *Pdgfb*^*wt/wt*^, *Pdgfb*^*wt/ret*^, *Pdgfb*^*ret/ret*^ animals. i.c.–intracerebral, i.p.–intraperitoneal, i.v.–intravenous. Quantification of number of vacuoles (B) and area in pixels covered by vacuoles (C) in cortex per field of view (FOV) in terminally sick *Pdgfb*^*wt/wt*^, *Pdgfb*^*wt/ret*^, *Pdgfb*^*ret/ret*^ mice showed similar extent of vacuolation (inoculation route—intravenous, dose—3 log LD_50_) (one-way ANOVA, Tukey’s multiple comparison test, p = 0.51 (B), p = 0.55 (C). Shown are means ±SD of biological replicates (N = 3–6). Scale bar: 10 μm.

**Fig 3:**
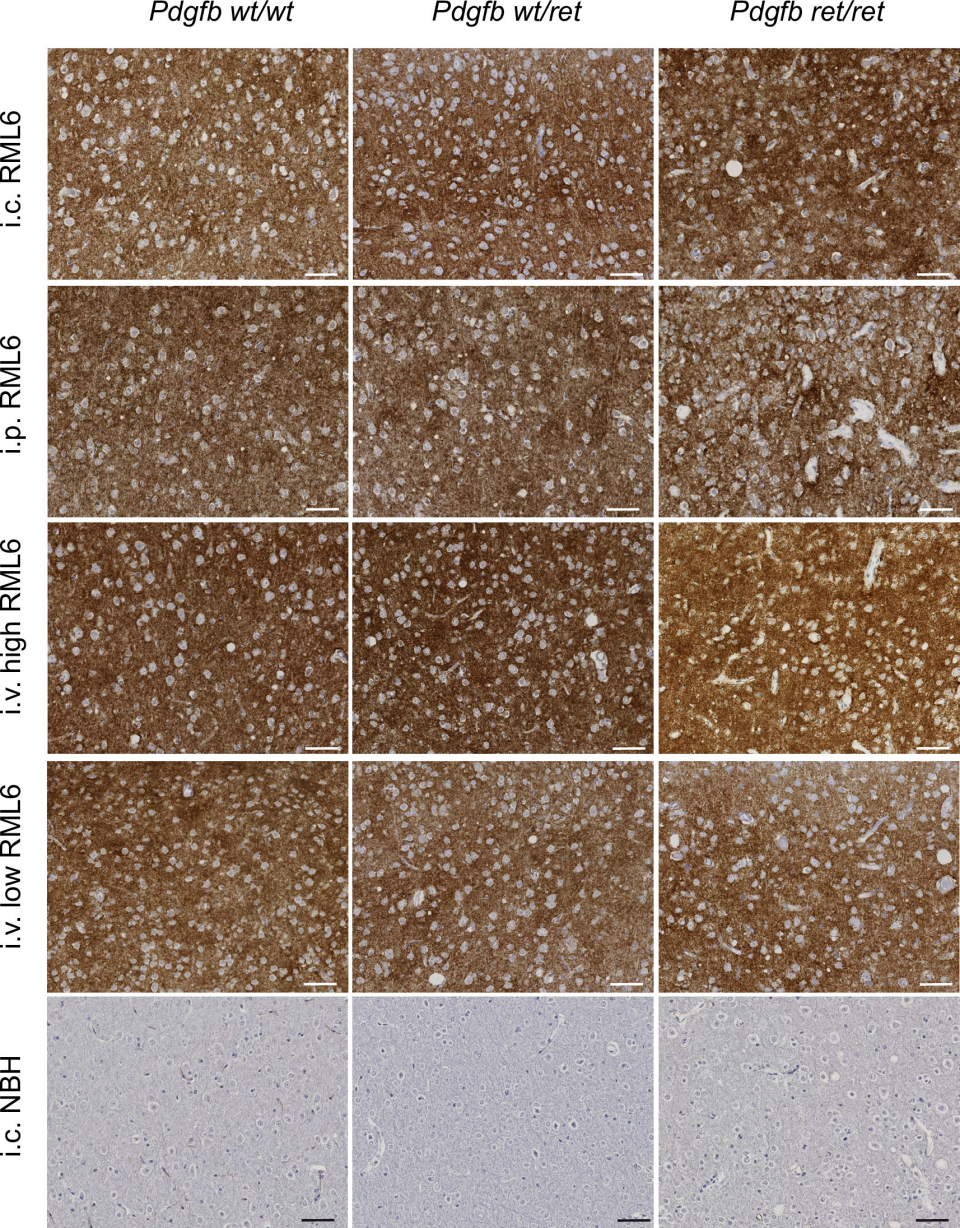
PrP^Sc^ deposition after RML6 inoculation in control and BBB-compromised mice. Brain sections (cerebral cortex) were stained for SAF84 to detect PrP^Sc^ deposits (dark brown) and co-stained with hematoxylin. Regardless of the inoculation route of RML6 prions, all animals (*Pdgfb*^*wt/wt*^, *Pdgfb*^*wt/ret*^, *Pdgfb*^*ret/ret*^) showed a similar extent of PrP^Sc^ deposition in their brains. No PrP^Sc^ deposits were detected in mice that received NBH (normal brain homogenate). i.c.–intracerebral, i.p.–intraperitoneal, i.v.–intravenous. Scale bar: 50 μm.

Inoculation of brain homogenate can induce autoimmune encephalitis, and it is not known whether such pathology may be exacerbated by a leaky BBB. Therefore, *Pdgfb*^*ret/ret*^ and control mice (*Pdgfb*^*wt/wt*^, *Pdgfb*^*wt/ret*^) were inoculated with non-infectious brain homogenate prepared from CD-1 strain as a control. None of these mice developed clinical signs of disease. They were sacrificed 350 days post-inoculation and showed no signs of encephalitis or prion pathology (Figs 2 and 3 and S2 and S5 Figs).

When prions are administered peripherally, neuroinvasion is dependent on peripheral replication in lymphoid tissues [4]. Therefore we assessed the course of prion disease in *Pdgfb*^*ret/ret*^ and control mice (*Pdgfb*^*wt/wt*^, *Pdgfb*^*wt/ret*^) after intraperitoneal administration of prions. Similarly to intracerebral inoculation, *Pdgfb*^*ret/ret*^ and control mice (*Pdgfb*^*wt/wt*^, *Pdgfb*^*wt/ret*^) showed no differences in prion disease pathogenesis after intraperitoneal inoculation (Fig 1B). Thus, we conclude that the altered BBB does not affect prion neuroinvasion when prions are administered peripherally nor lead to accelerated prion pathogenesis when administered intracerebrally.

It has been previously claimed that intravenously injected prions reach the CNS within minutes [7], and the authors of that study concluded that the quantity of PrP^Sc^ that reaches the brain via the BBB is sufficient to induce the disease in mice possessing a normal BBB. However, another study with Syrian hamsters found that although prions were found in the CNS few days after peripheral administration, the levels of prions were sub-infectious [21]. The mode of PrP^Sc^ transport into the CNS via brain endothelium is not known. The brain vasculature of *Pdgfb*^*ret/ret*^ mice is permeable to plasma proteins such as albumin, IgG, and the BBB permeability occurs at the level of endothelial transcytosis [10]. Normal brain endothelial cells show a paucity of transcytotic vesicles, however, the increased transcytosis is seen as a first sign of BBB defect in several brain insults (e.g. ischemic stroke) [22]. To directly assess the role of the open BBB in prion pathogenesis, *Pdgfb*^*ret/ret*^ and control mice (*Pdgfb*^*wt/wt*^, *Pdgfb*^*wt/ret*^) received RML6 intravenously, either a high dose (6 log LD_50_) or a low dose (3 log LD_50_). We reasoned that *Pdgfb*^*ret/ret*^ animals should show an earlier onset of prion disease compared to the control mice (*Pdgfb*^*wt/wt*^, *Pdgfb*^*wt/ret*^) since their BBB is permeable to plasma proteins. However, this was not the case, *Pdgfb*^*ret/ret*^ and control mice (*Pdgfb*^*wt/wt*^, *Pdgfb*^*wt/ret*^) animals did not show any statistically significant differences in the speed of prion pathogenesis and in their attack rate, either at high dose (6 log LD_50,_ p = 0.37) or at low dose (3 log LD_50_, p = 0.86) of intravenously administered prions (Fig 1C and 1D). The minimal trend towards earlier lethality of *Pdgfb*^*ret/ret*^ mice was well within the biological variability expected in this kind of experiments.

Our results using a mouse model with a compromised BBB due to increased transcytosis show that the permeability of CNS vasculature has a negligible effect on prion disease transmission into the CNS. This observation is in agreement with two decades of studies on the role of lymphoid organs in prion spread. If prions could enter the brain directly after peripheral inoculation, it would be difficult to understand why mice lacking B-cells [23] or complement components [24] experienced delayed neuroinvasion, and why the distance between follicular dendritic cells and peripheral nerve endings controls the speed of neuroinvasion [6]. Instead, the current results validate a model by which peripherally administered prions first colonize the lymphoid organs, then undergo a phase of clinically silent peripheral replication, and finally achieve neuroinvasion by exploiting peripheral nerves belonging to the sympathetic nervous system [5]. Therefore, prions resemble neurotropic viruses such as rhabdoviruses and herpesviruses that utilize retrograde axonal transport to gain access to the central nervous system, thereby bypassing the need for breaching the BBB. However, the limitation of our study is that the amount of PrP^Sc^ in the brain parenchyma shorty after intravenous injection with RML6 homogenate has not been quantified since currently available methods for detecting infectious prions in the brain parenchyma shortly after inoculations are not sufficient for this task.

Although *Pdgfb*^*ret/ret*^ and control mice (*Pdgfb*^*wt/wt*^, *Pdgfb*^*wt/ret*^) did not show differences in the prion disease incubation, they may conceivably differ with respect to the histological and biochemical hallmarks of prion infection. We therefore assessed the extent of spongiosis, the most characteristic feature of TSEs, on hematoxylin-eosin stained brain sections. All prion-inoculated animals, but none of the mice injected with NBH, showed the presence of spongiosis regardless of their genotype (Fig 2A). Quantification of vacuolation showed no difference in number of vacuoles and area covered by vacuoles between *Pdgfb*^*ret/ret*^ and control mice (*Pdgfb*^*wt/wt*^, *Pdgfb*^*wt/ret*^) (Fig 2B and 2C).

Microglia, whose activation in prion disease is considered neuroprotective partly by prion clearance [25, 26], express *Pdgfb* [27]. Although it is unlikely that the lack of Pdgfb retention motif will have a cell-autonomous effect on microglia—since microglia do not express the receptor of Pdgfb—*Pdgfrb*, we nevertheless assessed microgliosis in terminal stage of the disease. No differences in number of microglia in between *Pdgfb*^*ret/ret*^ and control mice (*Pdgfb*^*wt/wt*^, *Pdgfb*^*wt/ret*^) was detected (S3 Fig).

**Fig 4:**
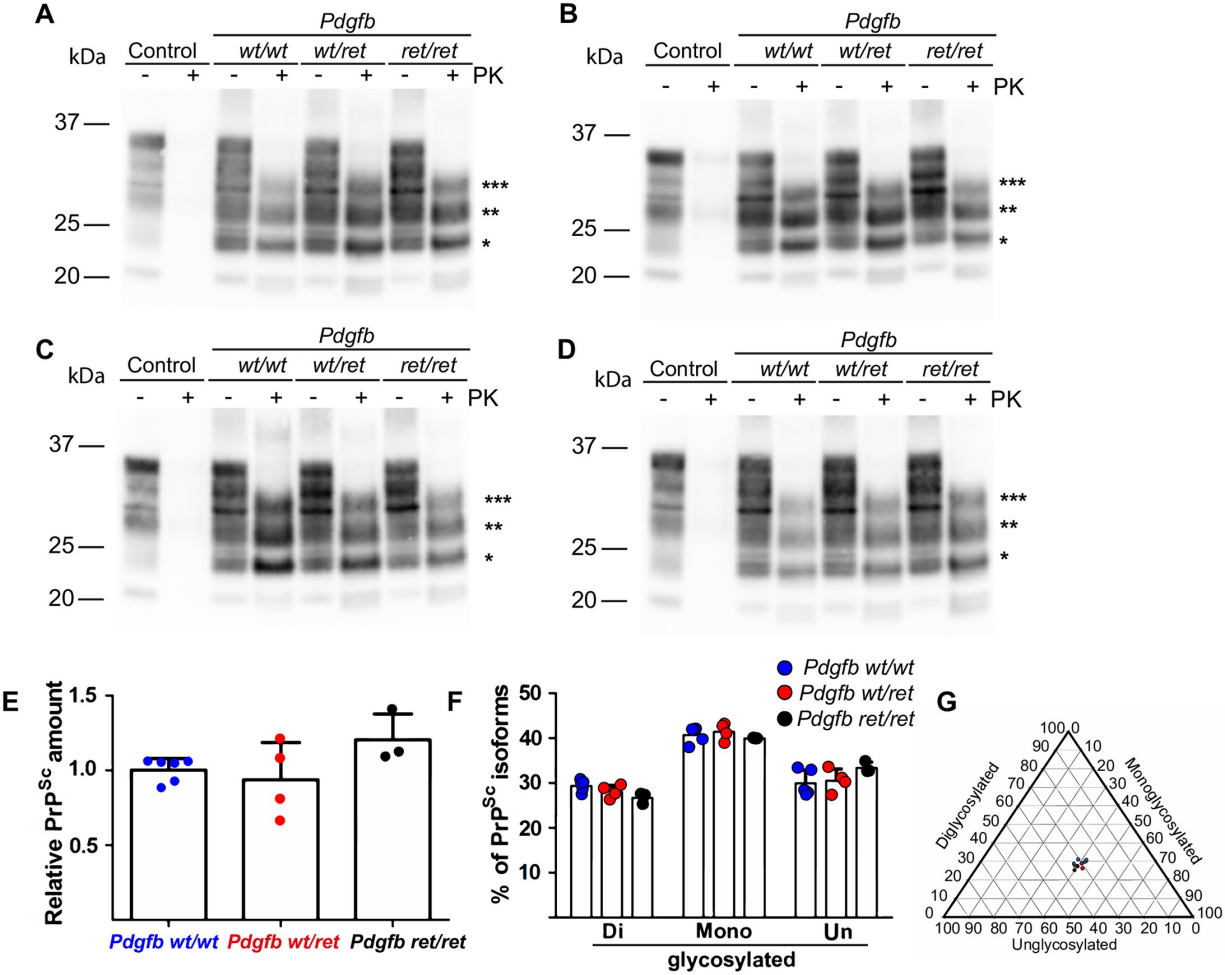
Western blotting and FRET analysis of brain homogenates from terminally sick *Pdgfb*^*wt/wt*^, *Pdgfb*^*wt/ret*^, *Pdgfb*^*ret/ret*^ animals inoculated with RLM6. PrP^C^ (control) is fully digested by proteinase K whereas PrP^Sc^ results in the exposure of 27–30 kDa protease-resistant fragments. + or - PK: with or without proteinase K digest. Intracerebrally (A), intraperitoneally (B), intravenously (C: 6 log LD_50_; D: 3 log LD_50_) inoculated control (*Pdgfb*^*wt/wt*^, *Pdgfb*^*wt/ret*^) and BBB-compromised (*Pdgfb*^*ret/ret*^) animals. Normal brain homogenate (NBH) inoculated animals were used as controls (Control). ***—di-glycosylated, **—mono-glycosylated and *—unglycosylated from of proteinase K-resistant PrP^Sc^. (E) Quantification of PrP^Sc^ in brain homogenates in animals inoculated intravenously with RML 6 (3 log LD_50_) using homogenous-FRET assay. The amount of proteinase K-resistant PrP^Sc^ in individual animals is expressed as a relative PrP^Sc^ amount compared to average of the wild-type mice (*Pdgfb*^*wt/wt*^). No difference in PrP^Sc^ amount is seen in terminally sick *Pdgfb*^*wt/wt*^, *Pdgfb*^*wt/ret*^, *Pdgfb*^*ret/ret*^ animals (one-way ANOVA, Tukey’s multiple comparison test, p = 0.15). Shown are means ±SD of biological replicates (N = 3–6). (F) The amount of PrP^Sc^ isoforms (expressed as percentage of total PrP^Sc^) did not differ between control (*Pdgfb*^*wt/wt*^, *Pdgfb*^*wt/ret*^) and *Pdgfb*^*ret/ret*^ animals (inoculation route—intravenous, dose—3 log LD_50_) (Two-way ANOVA, Bonferroni correction, p>0.05). Shown are mean ± SD of biological replicates (N = 3–6). (G) Tri-blot of glycoform percentages (see F) of PrP^Sc^ isoforms. Shown are biological replicates. *Pdgfb*^*wt/wt*^ are in blue, *Pdgfb*^*wt/ret*^ are in red and *Pdgfb*^*ret/ret*^ are in black. Tri-blot was generated in Excel using a template (Tri-blot v1.4.2) published by Graham and Midgley [30].

Finally, we investigated the levels of PrP^Sc^ accumulation using immunohistochemistry on formalin-fixed paraffin-embedded tissue sections, Western blotting and fluorescence-resonance energy transfer (FRET). Immunohistochemical analysis showed the presence of partially protease-resistant PrP (PrP^Sc^) in the brains of all prion-inoculated mice, regardless of the genotype and of the inoculation route, whereas NBH-injected mice never exhibited any PrP^Sc^ deposits (Fig 3). Interestingly, *Pdgfb*^*ret/ret*^ mice showed more prominent accumulation of PrP^Sc^ along blood vessels than wild-type mice (S4 Fig). All prion-inoculated animals showed PrP^Sc^ accumulation in spleen (S5 Fig). Western blotting of partially proteinase K-resistant PrP^Sc^ showed similar levels of disease-associated PrP among the three genotypes (*Pdgfb*^*wt/wt*^, *Pdgfb*^*wt/ret*^, *Pdgfb*^*ret/ret*^) for all inoculation routes and prion doses (Fig 4A–4D). Quantification of PrP^Sc^ levels using a FRET assay after PK digestion [18] did not show a difference in PrP^Sc^ levels between *Pdgfb*^*ret/ret*^ and control mice (*Pdgfb*^*wt/wt*^, *Pdgfb*^*wt/ret*^) (Fig 4E). It is conceivable that BBB opening would result in unorthodox prion replication and possibly in a strain shift [28]. Thus, we investigated whether differences in the BBB permeability in *Pdgfb*^*ret/ret*^ mice lead to alteration in prion strain. As a proxy for strain identification we quantified the ratio of the mono-, di-, and unglycosylated forms of PrP^Sc^. No significant difference between glycoform ratios were found among genotypes (Fig 4F and 4G), suggesting that no shift in prion strains had taken place in *Pdgfb*^*ret/ret*^ animals.

The altered BBB in *Pdgfb*^*ret/ret*^ mice in chronic inflammatory condition could lead to infiltration of peripheral leukocytes which could modify disease cause. However, other studies have shown that during prion disease there is a minimal recruitment of inflammatory monocytes and mice showing various T cell deficiencies develop clinical signs of prion disease with a comparable temporal dynamic/pattern to that seen in wild-type mice [23, 29]. The development of prion disease does not differ between *Pdgfb*^*ret/ret*^ and control mice (*Pdgfb*^*wt/wt*^, *Pdgfb*^*wt/ret*^) despite of the breached BBB of *Pdgfb*^*ret/ret*^ animals after all tested inoculation routes, including intracerebral inoculation. Thus, if there is a component in pathology caused by peripheral leukocytes then the effect on the disease has no detectable influence.

In conclusion, these data indicate that when prions are inoculated directly into the blood, the intactness of the BBB has a negligible effect on the incubation time of the disease. This suggests that extracerebrally administered prions do not need to trespass the BBB in order to enter the CNS. Alternatively, one could construe that prions can trespass the neurovascular barriers and colonize the brain through a mechanism that is fundamentally independent of the BBB. There is no factual evidence supporting the latter scenario.

A wealth of data accrued in multiple model systems supports the idea that prions, after entering the body from extraneural sites, undergo an early phase of replication in lymphoid organs, which is then followed by the colonization of peripheral nerve endings. It is then through the latter, according to this hypothesis, that prions eventually gain access to the CNS—akin to neurotropic viruses such as rhabdo- and herpesviruses. If prion spread to the CNS were directly hematogenic, one might expect the first site of CNS replication to be solely determined by the differential prion replication competence of select brain areas, rather than by the site of inoculation. In reality, however, the first site of CNS invasion after intraperitoneal inoculation with prions corresponds to the segmental projections of the peripheral nerves to the spinal cord [5, 6]. Moreover, chemical or immunological sympathectomy suffices to prevent neuroinvasion after intraperitoneal prion inoculation [5]. In the framework of the studies enumerated above, the data reported here add to the conjecture that prion spread from the periphery to the brain does not occur by direct transition across the BBB. Besides their significance for the basic understanding of prion neuroinvasion, these results may be of relevance to the possibility of developing effective post-exposure prophylaxis of prion diseases, which may prevent neurodegeneration even after extraneural infection has already taken place.

## Supporting information

S1 Fig Single horizontal image plane extracted from SPIM images of *Pdgfb*^*wt/ret*^ (A) and *Pdgfb*^*ret/ret*^ (B) animals demonstrating the entry of the 70 kDa-dextran Texas Red into the brain parenchyma in *Pdgfb*^*ret/ret*^ animals. The dynamic range of the *Pdgfb*^*wt/ret*^ image (**A**) was adjusted to show the background fluorescence emphasizing the lack of tracer in the brain. Excitation laser wavelength 594 nm, emission filtered with a 594 nm long pass filter. Optical slice thickness: 25 μm. Scale bar: 1 mm.
(PDF)

S2 Fig Prion histopathology after RML6 inoculation in control and BBB-compromised mice. Hematoxylin/eosin stained sections from the cerebella of prion-inoculated terminally sick (or control, NBH inoculated mice) *Pdgfb*^*wt/wt*^, *Pdgfb*^*wt/ret*^, *Pdgfb*^*ret/ret*^ animals showed similar extent of vacuolation. Scale bar: 100 μm.
(PDF)

S3 Fig The BBB-defect in *Pdgfb*^*ret/ret*^ animals did not alter microglia activation and number in terminally sick animals (inoculation route—Intravenous, dose—3 log LD_50_). **A**. Brain sections were stained for Iba1 to detect microglia in cortex and costained with hematoxylin. Scale bar: 100 μm. **B.** Quantification of Iba 1 positive cells in the cortex did not show a difference in microglia numbers between all tested genotypes (one-way ANOVA, Tukey’s multiple comparison test, p = 0.17) Shown are mean ±SD of biological replicates (N = 3–4).
(PDF)

S4 Fig PrP^Sc^ deposition after RML6 inoculation in control and BBB-compromised mice. Brain sections (corpus callosum) were stained for SAF84 to detect PrP^Sc^ deposits (dark brown) and co-stained with hematoxylin. Mice were intravenously inoculated (6 log LD_50_) with RML6. *Pdgfb*^*ret/ret*^ mice show conspicuous PrP^Sc^ deposits (arrows, B”) along the vasculature (arrow, A”). Such deposits were not visible in *Pdgfb*^*wt/wt*^ mice. Scale bars: 100 μm (A, B), 50 μm (A’, B’), 10 μm (A”, B”).
(PDF)

S5 Fig Splenic PrP^Sc^ deposition after RML6 inoculation in control and BBB-compromised mice. Spleen sections were stained for SAF84 to detect PrP^Sc^ deposits (dark brown) and co-stained with hematoxylin. Regardless of the inoculation route of RML6, all animals (*Pdgfb*^*wt/wt*^, *Pdgfb*^*wt/ret*^, *Pdgfb*^*ret/ret*^) showed PrP^Sc^ deposits in the spleen. No PrP^Sc^ deposits were detected in mice that received normal brain homogenate (NBH). Scale bar: 50 m.
(PDF)

S1 Movie Video based on SPIM recordings (594 nm excitation, 594 nm long pass filter) of *Pdgfb*^*ret/ret*^ mouse brain showing extravasated 70 kDa dextran-Texas Red. Start– 6 sec: presenting the 3D reconstruction of whole brain showing background fluorescence (594 nm excitation, 594 nm long pass filter); 7 sec– 16 sec: presenting z-stacks of optical sections showing dextran-Texas Red signal in red; 17 sec—end: a combined 3D reconstruction of whole brain (background autofluorescence combined with the signal from extravasated dextran Texas Red) demonstrates compromised BBB in the entire brain of *Pdgfb*^*ret/ret*^ mice. The most prominent BBB breakdown is seen in cerebral cortex.
(MP4)
