# Supplementary figures and images for: Prion pathogenesis is unaltered in a mouse strain with a permeable blood-brain barrier
Source: PLoS Pathog. 2018 Nov 29;14(11):e1007424. doi: 10.1371/journal.ppat.1007424 (PMC6264140; doi:10.1371/journal.ppat.1007424)

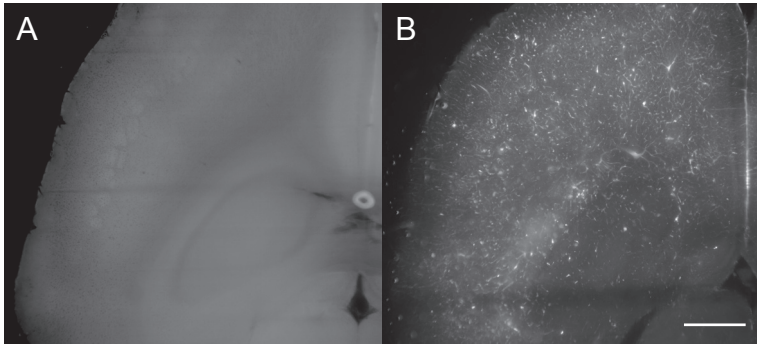

Supplement: S1 Fig — The dynamic range of the Pdgfbwt/ret image (A) was adjusted to show the background fluorescence emphasizing the lack of tracer in the brain. Excitation laser wavelength 594 nm, emission filtered with a 594 nm long pass filter. Optical slice thickness: 25 μm. Scale bar: 1 mm. (PDF) [file ppat.1007424.s001.pdf]

**A***Pdgfb wt/wt**Pdgfb wt/ret**Pdgfb ret/ret*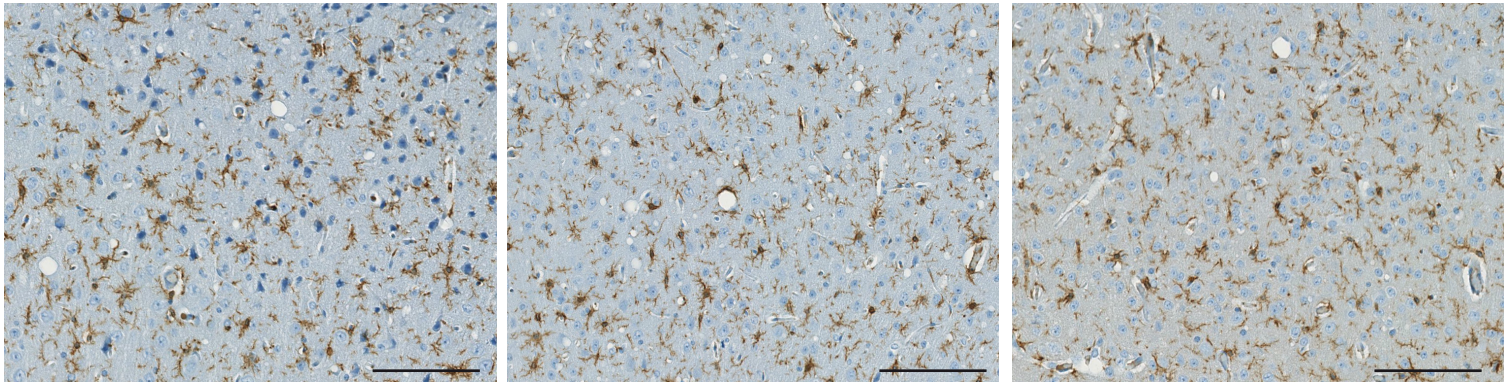**B**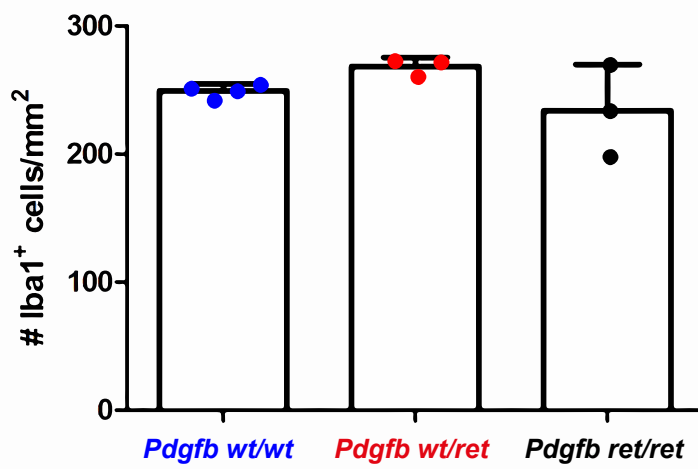

Supplement: S3 Fig — A. Brain sections were stained for Iba1 to detect microglia in cortex and costained with hematoxylin. Scale bar: 100 μm. B. Quantification of Iba 1 positive cells in the cortex did not show a difference in microglia numbers between all tested genotypes (one-way ANOVA, Tukey’s multiple comparison test, p = 0.17) Shown are mean ±SD of biological replicates (N = 3–4). (PDF) [file ppat.1007424.s003.pdf]

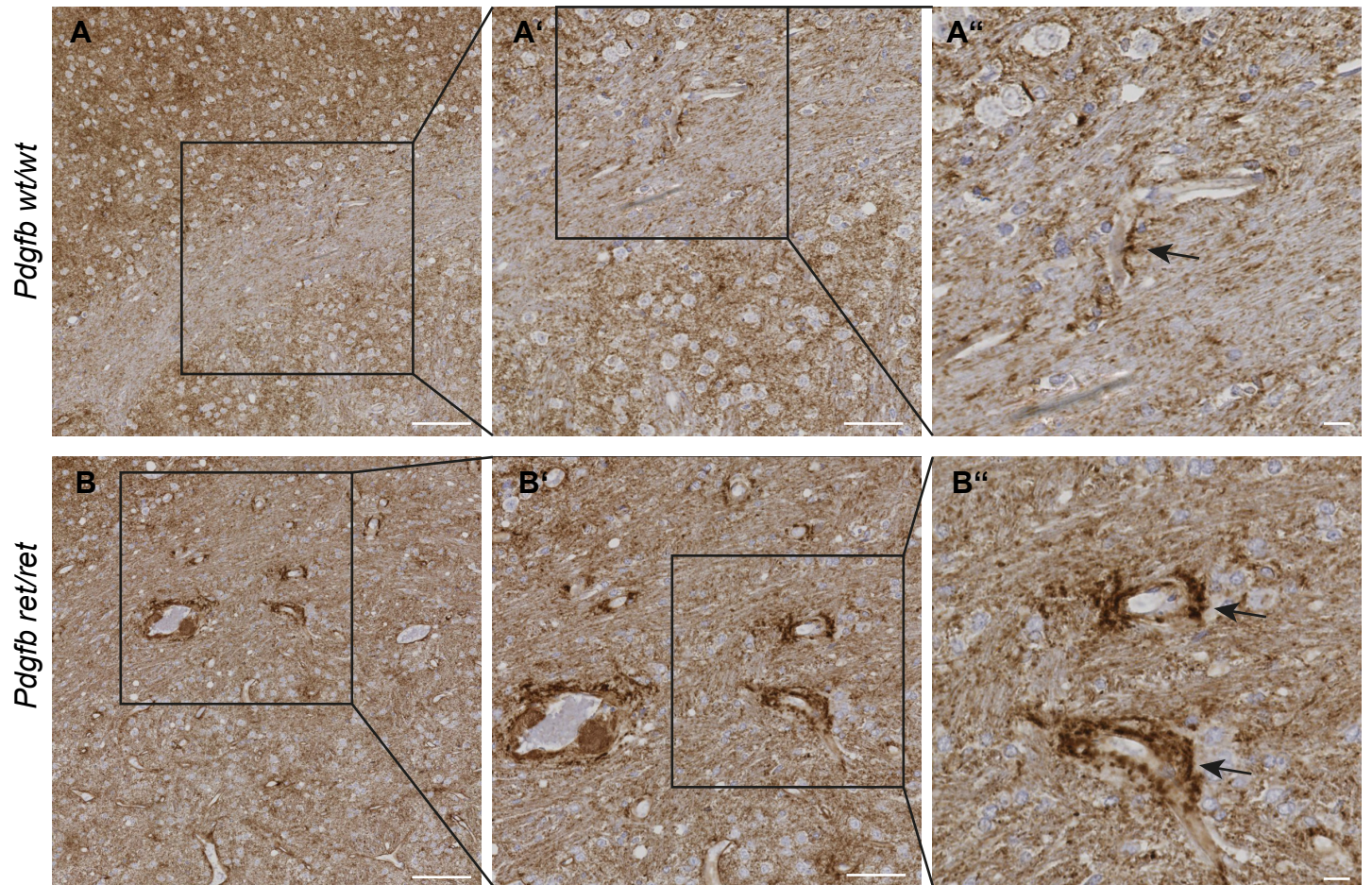

Supplement: S4 Fig — Brain sections (corpus callosum) were stained for SAF84 to detect PrPSc deposits (dark brown) and co-stained with hematoxylin. Mice were intravenously inoculated (6 log LD50) with RML6. Pdgfbret/ret mice show conspicuous PrPSc deposits (arrows, B”) along the vasculature (arrow, A”). Such deposits were not visible in Pdgfbwt/wt mice. Scale bars: 100 μm (A, B), 50 μm (A’, B’), 10 μm (A”, B”). (PDF) [file ppat.1007424.s004.pdf]
