# Supplementary material for: Prion pathogenesis is unaltered in a mouse strain with a permeable blood-brain barrier
Source: PLoS Pathog. 2018 Nov 29;14(11):e1007424. doi: 10.1371/journal.ppat.1007424 (PMC6264140; doi:10.1371/journal.ppat.1007424)
Supplement: S2 Fig — Hematoxylin/eosin stained sections from the cerebella of prion-inoculated terminally sick (or control, NBH inoculated mice) Pdgfbwt/wt, Pdgfbwt/ret, Pdgfbret/ret animals showed similar extent of vacuolation. Scale bar: 100 μm. (PDF) [file ppat.1007424.s002.pdf]

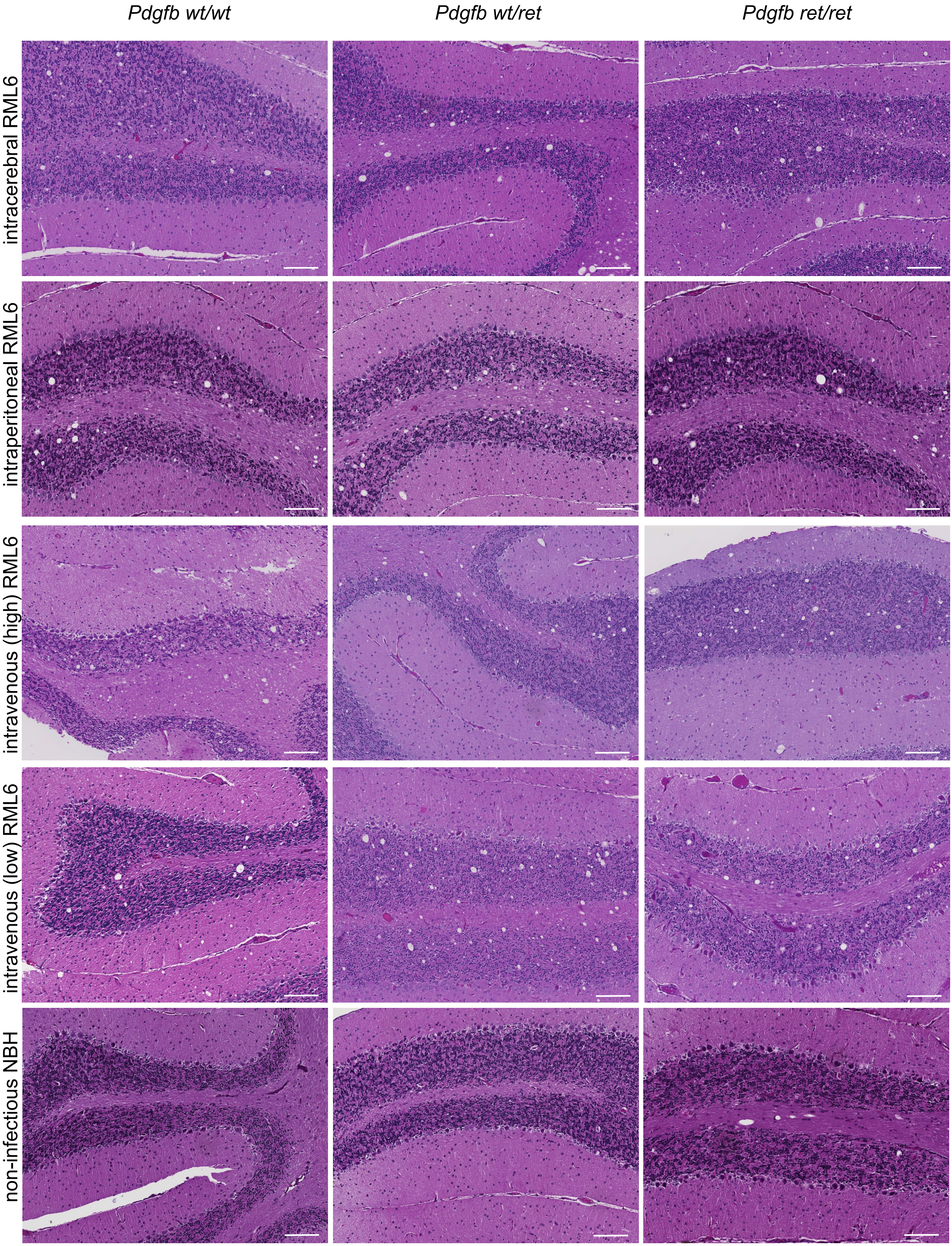

*Pdgfb wt/wt*
*Pdgfb wt/ret*
*Pdgfb ret/ret*
intracerebral RML6
intraperitoneal RML6
intravenous (high) RML6
intravenous (low) RML6
non-infectious NBH
