# Supplementary material for: Prion pathogenesis is unaltered in a mouse strain with a permeable blood-brain barrier
Source: PLoS Pathog. 2018 Nov 29;14(11):e1007424. doi: 10.1371/journal.ppat.1007424 (PMC6264140; doi:10.1371/journal.ppat.1007424)
Supplement: S5 Fig — Spleen sections were stained for SAF84 to detect PrPSc deposits (dark brown) and co-stained with hematoxylin. Regardless of the inoculation route of RML6, all animals (Pdgfbwt/wt, Pdgfbwt/ret, Pdgfbret/ret) showed PrPSc deposits in the spleen. No PrPSc deposits were detected in mice that received normal brain homogenate (NBH). Scale bar: 50 m. (PDF) [file ppat.1007424.s005.pdf]

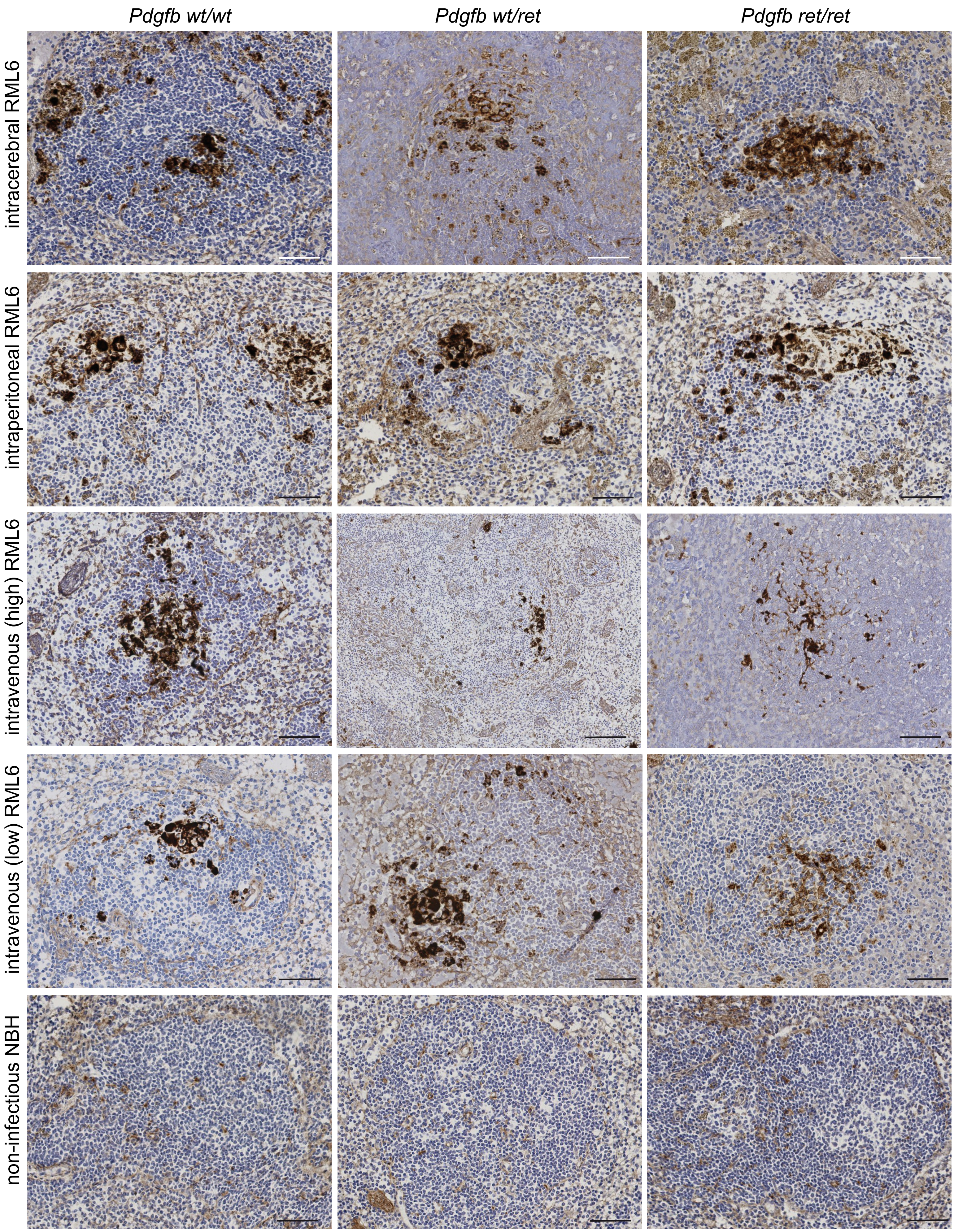
Pdgfb wt/wt
Pdgfb wt/ret
Pdgfb ret/ret
intracerebral RML6
intraperitoneal RML6
intravenous (high) RML6
intravenous (low) RML6
non-infectious NBH
